# LDLR is an entry receptor for Crimean-Congo hemorrhagic fever virus

**DOI:** 10.1038/s41422-023-00917-w

**Published:** 2024-01-05

**Authors:** Zhi-Sheng Xu, Wen-Tian Du, Su-Yun Wang, Mo-Yu Wang, Yi-Ning Yang, Yu-Hui Li, Zhen-Qi Li, Li-Xin Zhao, Yan Yang, Wei-Wei Luo, Yan-Yi Wang

**Affiliations:** 1 Wuhan Institute of Virology, Center for Biosafety Mega-science, Chinese Academy of Sciences, Wuhan, Hubei China; 2 Key Laboratory of Virology and Biosafety, Chinese Academy of Sciences, Wuhan, Hubei China; 3 University of Chinese Academy of Sciences, Beijing, China

**Keywords:** Cell biology, Molecular biology

## Abstract

Crimean-Congo hemorrhagic fever virus (CCHFV) is the most widespread tick-born zoonotic bunyavirus that causes severe hemorrhagic fever and death in humans. CCHFV enters the cell via clathrin-mediated endocytosis which is dependent on its surface glycoproteins. However, the cellular receptors that are required for CCHFV entry are unknown. Here we show that the low density lipoprotein receptor (LDLR) is an entry receptor for CCHFV. Genetic knockout of LDLR impairs viral infection in various CCHFV-susceptible human, monkey and mouse cells, which is restored upon reconstitution with ectopically-expressed LDLR. Mutagenesis studies indicate that the ligand binding domain (LBD) of LDLR is necessary for CCHFV infection. LDLR binds directly to CCHFV glycoprotein Gc with high affinity, which supports virus attachment and internalization into host cells. Consistently, a soluble sLDLR–Fc fusion protein or anti-LDLR blocking antibodies impair CCHFV infection into various susceptible cells. Furthermore, genetic knockout of LDLR or administration of an LDLR blocking antibody significantly reduces viral loads, pathological effects and death following CCHFV infection in mice. Our findings suggest that LDLR is an entry receptor for CCHFV and pharmacological targeting of LDLR may provide a strategy to prevent and treat Crimean-Congo hemorrhagic fever.

## Introduction

Crimean-Congo hemorrhagic fever virus (CCHFV) is a widely distributed tick-borne zoonotic virus of the *Orthonairovirus* genus in the *Nairoviridae* family of the *Bunyavirales* order, which has been reported in over 30 countries in Africa, Europe and Asia.^1,2^ Although CCHFV infection is asymptomatic in most vertebrates, it can result in severe viral hemorrhagic fever in humans with up to 30% fatality of diagnosed cases.^1,3,4^ Currently, there are no licensed vaccines or specific anti-CCHFV drugs, making the treatment options for CCHFV infection limited.^5^ Due to its great risk to public health and insufficient countermeasures, CCHFV has been continuously listed for years by WHO as a priority pathogen in research and development in public health emergency contexts.^6^

CCHFV possesses a negative sense tri-segmented RNA genome consisting of S, M and L, which encode the nucleoprotein (NP), glycoprotein precursor (GPC) and RNA-dependent RNA-polymerase (RdRP), respectively.^4,7^ The M-encoded GPC is co-translationally cleaved by cellular proteases to generate two structural glycoproteins, Gc and Gn, and three non-structural proteins Mucin, GP38 and NSm.^8–10^ The Gc and Gn glycoproteins form a locally ordered lattice of heterodimers on the viral surface, which are responsible for binding to cellular receptors and subsequent fusion of the viral envelope with host cellular membranes.^11–13^ So far, Gc is the only known target of CCHFV-neutralizing antibodies.^12–15^ It has been reported that Gc stays as monomeric in the prefusion state and turns to trimeric to drive membrane fusion in the acidic environment. The entry of CCHFV into target cells is via receptor-mediated endocytosis and multivesicular bodies are the sites of virus-endosome membrane fusion.^13,16–18^ However, the cellular receptor(s) for CCHFV infection remains unknown, which greatly hampers the understanding of CCHFV–host interaction and development of effective treatment strategies for CCHF. In this study, we identified the low density lipoprotein receptor (LDLR) as a critical entry receptor for infection of CCHFV. Using biochemical, cellular and genetic approaches, we demonstrate that CCHFV Gc directly binds to LDLR, which can fully mediate its entry into various cells from mouse to human origin as well as establishment of successful infection and pathogenesis in mice.

## Results

### Identification of LDLR as a candidate host factor for CCHFV infection

**Fig. 1:**
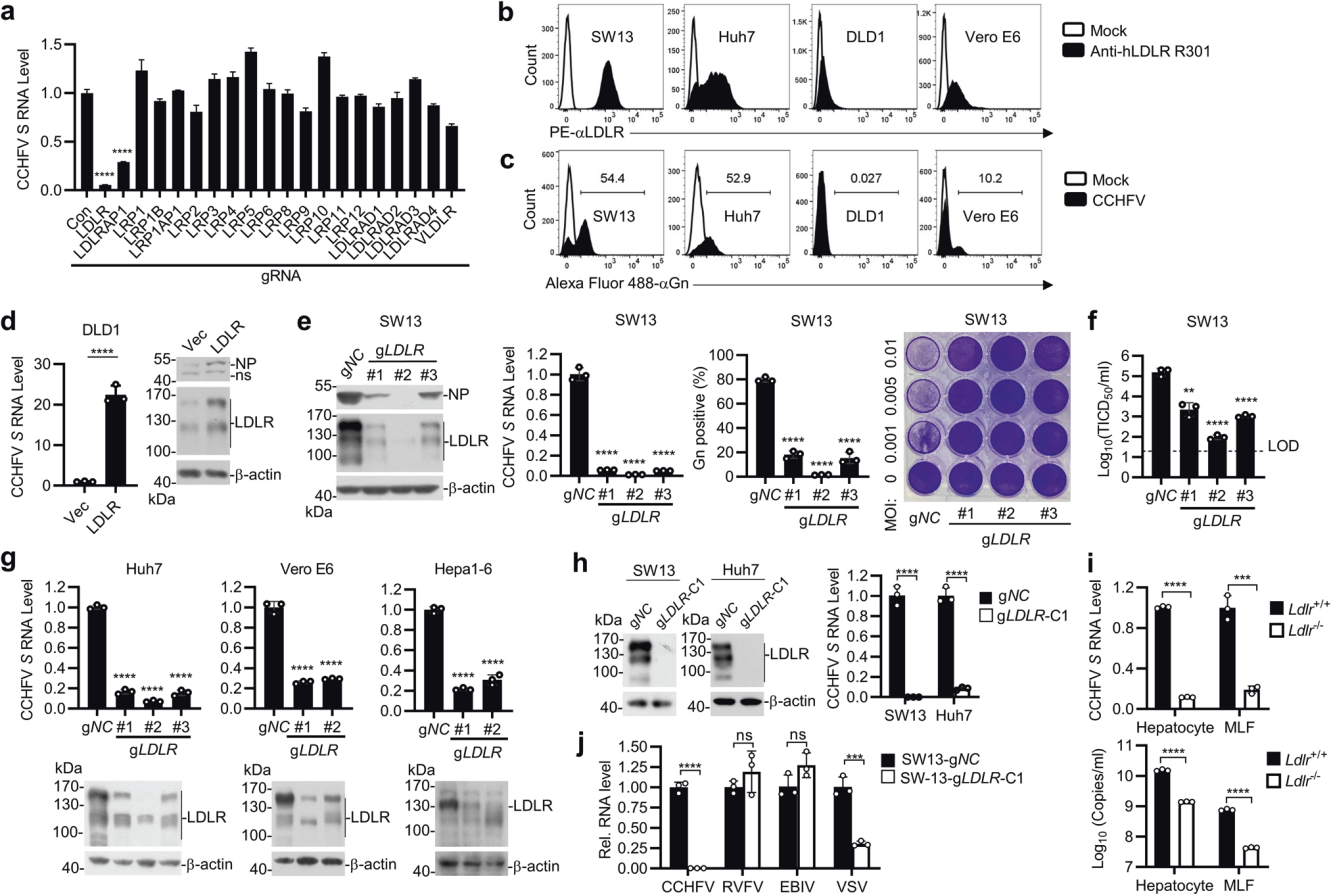
LDLR is an important host factor for CCHFV infection. **a** Screening of LDLR and LDLR-related proteins (LRPs) that are crucial for CCHFV infection. The HEK293T cells were edited with a control or sgRNAs targeting the genes encoding LDLR and LRPs (two sgRNAs for each gene). After puromycin selection, the cell pools were infected with CCHFV (MOI = 0.05) for 24 h before RT-qPCR was performed. Data were normalized to the relative mRNA level of CCHFV *S* in the control sgRNA-edited cells. **b** Surface expression of LDLR in different cell lines. The indicated cell lines were assessed by flow cytometry using the anti-LDLR mAb (R301). **c** CCHFV infectivity of different cell lines. The indicated cell lines were infected with CCHFV (MOI = 0.05) for 48 h. The CCHFV Gn-positive cells were examined by flow cytometry with a customized anti-Gn monoclonal antibody (7A11). **d** Overexpression of LDLR enhances CCHFV infection in DLD1 cells. Control and LDLR-overexpressing DLD1 cells were infected with CCHFV (MOI = 0.05) for 24 h (left panel) or 48 h (right panel). The levels of CCHFV *S* mRNA and NP protein were determined by RT-qPCR (left panel) and immunoblots (right panel), respectively. **e** Effects of LDLR-deficiency on CCHFV infection in SW13 cells. SW13 cells were edited with a control (g*NC*) or three individual sgRNAs targeting different regions of *LDLR* coding sequence (g*LDLR*). The control and *LDLR* sgRNA-edited SW13 cell pools were infected with CCHFV (MOI = 0.05). CCHFV NP expression (left panel, 48 hpi), mRNA level of CCHFV *S* segment (2^nd^ panel, 24 hpi), percentage of Gn-positive cells (3^rd^ panel, 48 hpi) and cell cytopathic effects (right panel, 72 hpi) was measured by immunoblots, RT-qPCR, flow cytometry and crystal violet staining, respectively. For bar graphs, data are normalized to that of the control gRNA-edited cells. **f** Effects of LDLR-deficiency on production of progeny viruses. SW13 cells were edited with a control (g*NC*) or three individual sgRNAs targeting different regions of *LDLR* coding sequence (g*LDLR*). The sgRNA-edited SW13 cell pools were then infected with CCHFV (MOI = 0.05) for 72 h. Titers of progeny viruses in the supernatants were measured by TCID_50_ assay. Data are normalized to that of the control gRNA-edited cells. LOD, limit of detection. **g** Effects of LDLR-deficiency on CCHFV infection in various cells. Huh7, Vero E6 and Hepa1-6 cells were edited with a control gRNA or the indicated numbers of gRNAs targeting *LDLR* gene. Cells were infected with CCHFV (MOI = 0.05) for 24 h before RT-qPCR was performed. Data are normalized to the CCHFV *S* mRNA level in the control gRNA-edited cells. **h** CCHFV infectivity in LDLR-knockout SW13 and Huh7 cells. Single clones of LDLR-knockout SW13 and Huh7 were isolated and confirmed by immunoblots (left). The control (g*NC*) or LDLR-deficient clone (g*LDLR*-C1) were infected with CCHFV (MOI = 0.05) for 24 h before RT-qPCR analysis. Data are normalized to that of each control gRNA-edited cells. **i** CCHFV infectivity in *Ldlr*^*−/−*^ primary cells. Primary hepatocytes and lung fibroblasts (MLFs) prepared from WT and *Ldlr*^*−/−*^ mice were incubated with CCHFV (MOI = 0.05). The mRNA level of CCHFV *S* segment (top, 48 hpi) and the viral genomic copies in the supernatant (bottom, 72 hpi) were measured by RT-qPCR. **j** Effects of LDLR-deficiency on CCHFV, RVFV, EBIV and VSV infection. The control (g*NC*) or LDLR-deficient clone (g*LDLR*-C1) were inoculated with the indicated viruses for 24 h before RT-qPCR was performed. Data are represented as mean ± SD. **P < 0.01; ***P < 0.001; ****P < 0.0001.

Early studies have shown that CCHFV prefers basolateral entry in polarized epithelial cells, such as Madin-Darby canine kidney 1 (MDCK-1) cells and human colorectal adenocarcinoma Caco-2 cells,^19,20^ which is similar to Vesicular Stomatitis Virus (VSV).^21,22^ LDLR has been identified as a major entry receptor of VSV and other LDLR related proteins (LRPs) serve as alternative receptors.^23,24^ It has also been shown that the level of LDLR on the basolateral surface of MDCK cells is dramatically higher than that on their apical surface.^23,24^ This prompts us to investigate whether LDLR and its family members play a role in CCHFV infection. Using the CRISPR-Cas9 system to target two independent sites of each gene,^25^ we generated 293 T cells deficient of LDLR family members. These edited cell lines were inoculated with CCHFV (MOI = 0.05) for 24 h and then expression of viral genomic *S* segment was examined by reverse transcription-quantitative PCR (RT-qPCR). As shown in Fig. 1a, knockout of LDLR and LDLR adapter protein 1 (LDLRAP1), but not the other 18 LDLR family members or related signaling components, dramatically inhibited the mRNA level of *S* segment upon CCHFV infection (Fig. 1a). These results suggest that LDLR and LALRAP1 but not the other examined proteins are important for CCHFV infection.

### The levels of LDLR are correlated to CCHFV infectivity

LDLR is a cellular membrane glycoprotein that functions in the binding and internalization of circulating cholesterol-containing lipoprotein particles, whereas LDLRAP1 is a cytosolic adapter protein that interacts with the cytoplasmic tail of LDLR and facilitates its endocytosis.^26^ Therefore, we then focused on LDLR and attempted to determine whether it is a cellular entry receptor for CCHFV. We firstly analyzed the relevance between LDLR expression and CCHFV infectivity using a panel of cell lines, including the human adrenal cortical carcinoma SW13, the human liver cancer Huh7, the human colorectal cancer DLD1 cells and the monkey kidney Vero E6 cells. Flow cytometric analysis of membrane LDLR level with an anti-LDLR monoclonal antibody (R301) indicated that LDLR was highly expressed in SW13 and Huh7, moderately expressed in Vero E6 cells, and barely detectable in DLD1 cells (Fig. 1b). Correspondingly, after CCHFV infection, flow cytometric analysis using an customized monoclonal antibody against CCHFV Gn (7A11) indicated that the percentage of infected cells was high in SW13 and Huh7 cells (> 50%), moderate in Vero E6 cells (~10%) and almost undetectable in DLD1 cells (0.027%) (Fig. 1c). Ectopic expression of LDLR in DLD1 cells significantly enhanced CCHFV infection as demonstrated by increased CCHFV *S* mRNA and NP protein (Fig. 1d). These results suggest that membrane LDLR levels are positively correlated to the infectivity of CCHFV.

### Deficiency of LDLR impairs CCHFV infectivity in divergent cell types

Since SW13 cell line expresses high level of LDLR and is highly susceptible to CCHFV infection, we further edited LDLR in these cells by CRISPR-Cas9 with three individual sgRNAs that target different sites in the LDLR coding sequence to confirm its function on CCHFV infection. As shown in Fig. 1e, CCHFV infectivity was impaired in *LDLR*-edited SW13 cells as determined by the dramatically reduced level of CCHFV NP protein, mRNA level of *S* segment, percentage of Gn-positive cells and cytopathic effects of infected cells. Consistently, the titers of progeny viruses in the cell culture supernatant were also decreased in the *LDLR*-edited SW13 cells (Fig. 1f). In these experiments, the inhibitory degrees of CCHFV infectivity were correlated with the knockdown efficiencies of the three LDLR sgRNAs (Fig. 1e). These results suggest that LDLR is an important cellular factor for CCHFV. We further confirmed the function of LDLR in CCHFV infection in Huh7, Vero E6 and the murine hepatocellular carcinoma Hepa1-6 cells by editing *LDLR* with two or three different sgRNAs. Similarly, LDLR-knockdown impaired CCHFV infection as indicated by dramatically reduced levels of *S* segment mRNA in all examined cell lines (Fig. 1g), suggesting that LDLR plays a conserved role in mediating CCHFV infection in human, monkey and mouse cells. We further aligned the amino acid sequences of the ligand binding domain (LBD) of LDLR across different species including human, monkey, mouse, rat, hamster, rabbit, hedgehog, goat, sheep, cattle, horse and camel, and the results indicated that the similarity of human LDLR LBD to those of other mammalian orthologs is 80–96% (Supplementary information, Fig. S1), demonstrating a high homology of LDLR among mammals.

In the above experiments, we used *LDLR*-edited cell pools in which LDLR expression was dramatically reduced but not completely abolished. In these cells, CCHFV infection was also greatly reduced but not completely abrogated. To determine whether the remaining infectivity is caused by the remaining expression of LDLR or by a redundant cellular factor, we isolated single clones of *LDLR*-edited SW13 and Huh7 cells in which LDLR expression was completely deficient and infected these cells with CCHFV (Fig. 1h). The results indicated that CCHFV infection was completely abrogated in LDLR-deficient SW13 cells, but ~10% infectivity remained in LDLR-deficient Huh7 cells as determined by the level of CCHFV *S* mRNA (Fig. 1h). Furthermore, we examined the infectivity of CCHFV in primary hepatocytes and lung fibroblasts (MLFs) from WT and *Ldlr*^*−/−*^ *mice*. The results confirmed that mRNA level of CCHFV *S* segment and production of progeny viruses were significantly reduced in LDLR-deficient cells, though low level of infectivity still remained (Fig. 1i). These results suggest that LDLR is required for infection of CCHFV in SW13 cells, while a cellular factor other than LDLR may support CCHFV infection with low efficiency in Huh7 cells and the primary mouse hepatocytes and MLFs.

Therefore, we tested whether other LRPs function redundantly with LDLR to facilitate CCHFV infection. To do this, we used siRNAs to knockdown the 18 known LRPs in LDLR-deficient Huh7 cells and examined CCHFV infectivity in these cells (Supplementary information, Fig. S2a). In addition, we also ectopically expressed 15 LRPs in DLD1 cells and examined their effects on CCHFV infectivity (Supplementary information, Fig. S2b). The results indicated that none of the examined LRPs can function as a cellular factor to support CCHFV infection, which should impair CCHFV infection when depleted by siRNA in LDLR-deficient Huh7 cells and/or promote CCHFV infection when overexpressed in DLD1 cells. Moreover, preincubation of LDLR-deficient Huh7 cells with soluble human receptor-associated protein (RAP), a common chaperone that can block ligand binding to all LRP family members except LDLR,^27^ also had no marked effects on CCHFV infection (Supplementary information, Fig. S2c). In similar experiments, RAP could completely abolish infection of Semliki Forest virus (SFV) in Huh7 cells (Supplementary information, Fig. S2c), which has been shown to utilize VLDLR and ApoER2 (also known as LRP8) of the LRP family as entry receptors.^28^ Taken together, these results suggest that LDLR but not the known LRPs is essential for CCHFV infection.

We next asked whether LDLR is required for infection of other bunyaviruses, including Rift Valley Fever Virus (RVFV) and Ebinur Lake Virus (EBIV), by examination of their infectivity in LDLR-deficient SW13 cells. The results indicated that LDLR-deficiency had no marked effects on infection of RVFV or EBIV in SW13 cells (Fig. 1j). In the same experiments, LDLR-deficiency impaired infection of VSV, which is consistent with a previous report that LDLR is a receptor for VSV.^23^ Collectively, these results suggest that LDLR is specifically required for infection of CCHFV but not the other examined bunyaviruses in SW13 cells.

### The ligand-binding domain of LDLR is required for CCHFV infection

**Fig. 2:**
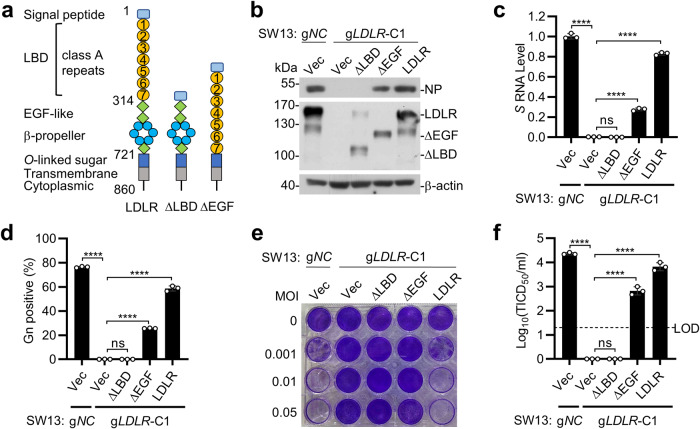
LDLR is required for CCHFV infection. **a** A schematic presentation of full-length LDLR and its truncated mutants that lack the ligand binding domain (∆LBD) and epidermal growth factor like domain (∆EGF). **b–f** The LBD of LDLR is important for CCHFV infection. The control (g*NC*) or LDLR-deficient (g*LDLR*-C1) SW13 cells were reconstituted with a control vector, full-length LDLR or the indicated LDLR truncations respectively. The cells were inoculated with CCHFV (MOI = 0.05), and CCHFV NP expression (**b** 48 hpi), mRNA level of CCHFV *S* segment (**c** 24 hpi), percentage of CCHFV Gn-positive cells (**d** 48 hpi), cell survival (**e** 72 hpi) and production of CCHFV progeny viruses (**f** 72 hpi) were measured by RT-qPCR, immunoblots, flow cytometry, crystal violet staining and TCID_50_ assay, respectively. LOD, limit of detection. Data are represented as mean ± SD. ****P < 0.0001; ns, not significant.

Because LDLR is a membrane protein localized on the cell surface, we hypothesized that LDLR functions as an entry receptor of CCHFV. We firstly investigated whether the extracellular domain of LDLR is required for CCHFV infection. LDLR contains a signal peptide, seven LDLR type A repeats in the LBD, immediately followed by an EGF-like domain containing a β-propeller module, a membrane-proximal O-linked sugar domain, a transmembrane anchor, and a cytoplasmic domain (Fig. 2a). We reconstituted the LDLR-deficient SW13 cells with full-length LDLR and two truncated mutants which lack the LBD (∆LBD) or EGF-like domain (∆EGF) respectively (Fig. 2a). CCHFV infection of these reconstituted cells showed that the full-length LDLR restored CCHFV infectivity as determined by comparable levels of NP expression (Fig. 2b), *S* segment mRNA (Fig. 2c), percentage of Gn-positive cells (Fig. 2d), cytopathic effects (Fig. 2e) and progeny virus production (Fig. 2f) with control edited SW13 cells which express endogenous LDLR. In these experiments, reconstitution of LDLR-deficient SW13 cells with LDLR(∆EGF) partially supported CCHFV infection whereas reconstitution with LDLR(∆LBD) failed to support CCHFV infection (Fig. 2b–f). These results suggest that the LBD of LDLR is required for LDLR-mediated infection of CCHFV.

### LDLR is essential for CCHFV binding to cells

**Fig. 3:**
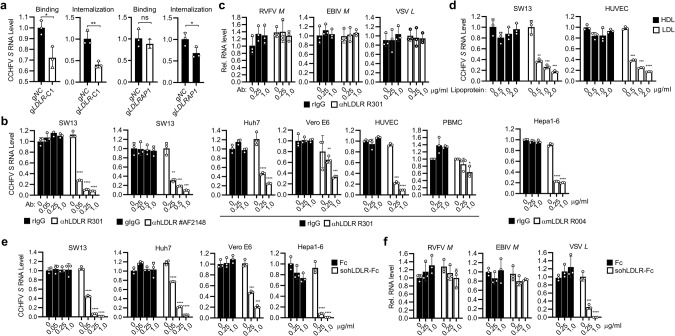
LDLR is essential for CCHFV binding to cells. **a** Effects of LDLR on CCHFV attachment and internalization. The control (g*NC*), LDLR-deficient (g*LDLR*-C1) or LDLRAP1-deficient (g*LDLRAP1*) SW13 cells were incubated with CCHFV at 4 °C for 1 h (for binding assay), or followed with incubation at 37 °C for 1 h (for internalization assay). The cells were collected and CCHFV *S* mRNA level was measured by RT-qPCR. Data are normalized to the CCHFV *S* mRNA level in the control gRNA-edited cells. **b** Effects of LDLR blocking antibodies on CCHFV infection. SW13, Huh7, Vero E6, primary human PBMCs and HUVECs, and mouse Hepa1-6 cells were pre-incubated with a rabbit anti-hLDLR mAb (R301), a goat anti-hLDLR pAb (#AF2148), a rabbit anti-mLDLR mAb (R004), or their respective control IgGs as indicated for 1 h before CCHFV infection (MOI = 0.05). Twenty-four hours after infection, the cells were collected for RT-qPCR analysis for CCHFV *S* mRNA level. Data are normalized to that of cells treated with the respective control IgG at 0 μg/mL. **c** Effects of LDLR blocking antibodies on the entry of RVFV, EBIV and VSV. SW13 cells were pre-incubated with the indicated concentrations of a control rIgG or a rabbit anti-hLDLR mAb (R301) for 1 h before infection RVFV, EBIV or VSV. Twenty-four hours after infection, mRNA levels of RVFV *M* segment, EBIV *S* segment, or VSV *L* gene were measured by RT-qPCR analysis. Data are normalized to that of cells treated with the respective control IgG at 0 μg/mL. **d** LDL inhibits CCHFV infection. SW13 cells (left) and HUVEC cells (right) were pre-treated with the indicated concentrations of LDL for 1 h and then left uninfected or infected with CCHFV (MOI = 0.05). Twenty-four hours post infection, CCHFV *S* mRNA level was analyzed by RT-qPCR. Data are normalized to that of CCHFV infected cells without LDL treatment. **e** The soluble human LDLR-Fc fustion protein (sohLDLR-Fc) inhibits CCHFV infection. CCHFV (MOI = 0.05) was pre-incubated with the indicated concentrations of Fc or sohLDLR-Fc for 1 h before infection of SW13 and Huh7 cells. Twenty-four hours post infection, CCHFV *S* mRNA level was analyzed by RT-qPCR. Data are normalized to that of cells infected with un-pretreated viruses. **f** Effects of sohLDLR-Fc on RVFV, EBIV and VSV infection in SW13 cells. RVFV (MOI = 0.1), EBIV (MOI = 0.5) or VSV (MOI = 0.1) were pre-incubated with the indicated concentrations of sohLDLR-Fc or Fc for 1 h before infection of SW13 cells. Twenty-four hours post infection, mRNA levels of RVFV *M* segment, EBIV *S* segment, or VSV *L* gene were measured by RT-qPCR analysis. Data are normalized to that of cells infected with the respective un-pretreated viruses. Data are represented as mean ± SD. *P < 0.05; **P < 0.01; ***P < 0.001; ****P < 0.0001.

We then investigated whether LDLR is required for CCHFV binding to the cell surface and its internalization. We found that the binding of CCHFV at 4 °C to as well as the internalization of CCHFV at 37 °C in LDLR-deficient SW13 cells was significantly reduced in comparison with the control cells (Fig. 3a, left 2 panels). In similar experiments, internalization of CCHFV but not its binding to cell surface was significantly affected in LDLRAP1-deficient cells (Fig. 3a, right 2 panels), which is an adapter protein involved in LDLR internalization upon its ligand binding.^26^ In these experiments, there were residual CCHFV binding and internalization in LDLR-deficient cells, which may be caused by nonspecific binding of CCHFV to glycans, cell adhesion molecules and other proteins on the cell surface as most viruses do. Nevertheless, these results suggest that LDLR is necessary for optimal cellular binding and internalization of CCHFV. To further corroborate an effect of LDLR on binding and entry of CCHFV, we investigated whether specific antibodies against LDLR could block CCHFV infection. Pre-treatment of SW13 cells with a monoclonal anti-human LDLR antibody (R301) or a polyclonal anti-human LDLR antibody (#AF2148) reduced CCHFV infection in a dose-dependent manner (Fig. 3b). Pre-treatment with the R301 mAb also dose-dependently reduced CCHFV infection in Huh7, Vero E6 and primary human umbilical vein endothelial cells (HUVECs) while had limited inhibition of CCHFV infection in primary human PBMCs (Fig. 3b). Additionally, pre-treatment with a monoclonal antibody against mouse LDLR (R004) markedly reduced CCHFV infection in mouse Hepa1-6 cells (Fig. 3b). In similar experiments, R301 mAb pre-treatment had no obvious effects on RVFV, EBIV or VSV infection in SW13 cells (Fig. 3c). We also used LDL, the ligand for LDLR, as well as an Fc fusion protein with the soluble extracellular domains of human LDLR (sohLDLR-Fc) (Supplementary information, Fig. S3a) to evaluate whether they could inhibit CCHFV infection. Pre-incubation of SW13 and HUVEC cells with LDL inhibited CCHFV infection dose-dependently (Fig. 3d). In addition, pre-incubation of CCHFV with sohLDLR-Fc, but not the control Fc, reduced viral infection in SW13, Huh7, Vero E6 and Hepa1-6 cells in a dose-dependent manner (Fig. 3e). In addition, pre-incubation with sohLDLR-Fc also reduced VSV, but not RVFV or EBIV infection (Fig. 3f). Collectively, these results suggest that LDLR mediates CCHFV entry.

### LDLR binds directly to Gc of CCHFV

**Fig. 4:**
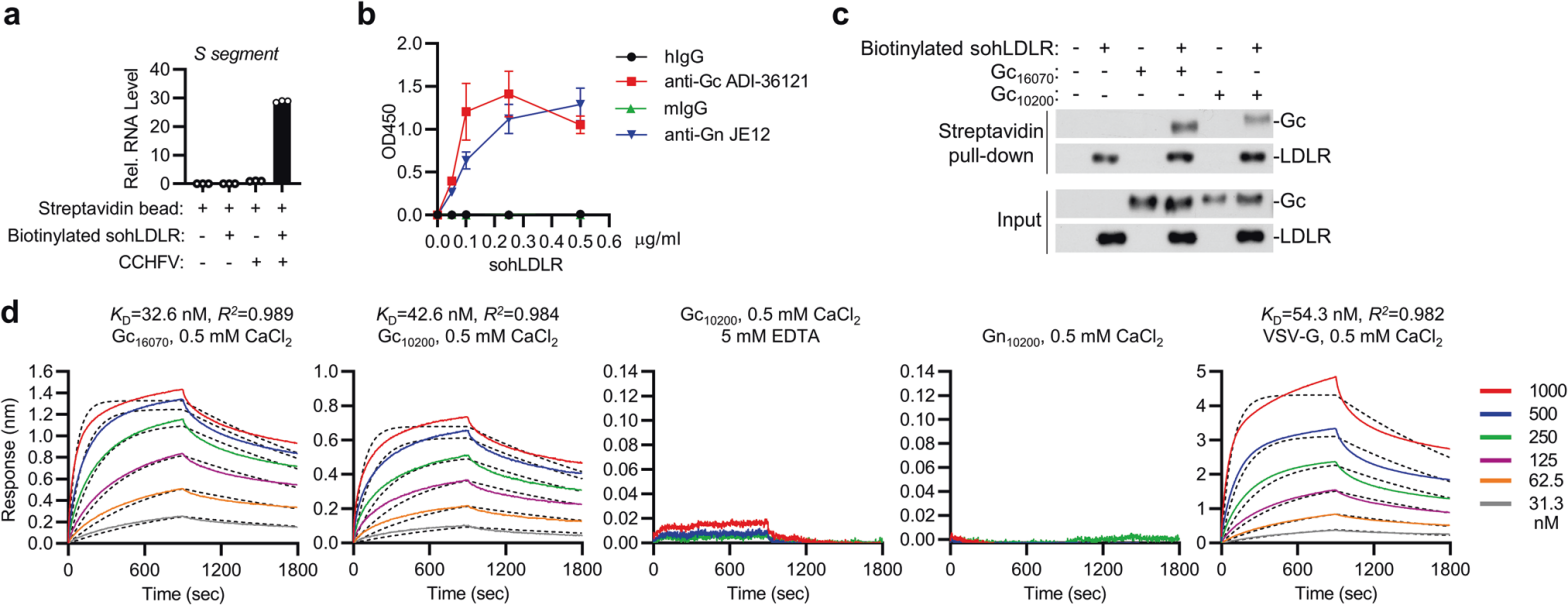
LDLR binds directly to Gc of CCHFV. **a** Pull-down of CCHFV virions by sohLDLR. CCHFV, biotinylated sohLDLR and magnetic streptavidin beads were co-incubated as indicated. Pull-down was performed with a magnet and the pellet was subjected to RT-qPCR analysis. Data are represented as mean ± SD. **b** Monoclonal antibodies against CCHFV Gc (ADI 36121), Gn (JE12) or their respective control IgG was immobilized on plates. ELISA-based binding assays were performed with CCHFV, biotinylated sohLDLR and Avidin-HRP. Data are represented as mean ± SD. **c** Recombinant Gc of CCHFV YL16070 and IbAr 10200 strains and biotinylated sohLDLR were co-incubated as indicated. Pull-down assay was performed with magnetic streptavidin beads and the pellets were subjected to immunoblots with the indicated antibodies. **d** Biotinylated sohLDLR was immobilized onto the streptavidin biosensors. Binding parameters of recombinant Gc or Gn of the YL16070 and IbAr 10200 strain and VSV-G to LDLR were measured by Bio-Layer Interferometry (BLI) in the indicated PBS buffers. Fitted curves are shown with dotted lines. A 1:1 binding model was used to calculate the *K*_D_.

We next determined whether LDLR could directly bind to CCHFV virions. We incubated CCHFV with biotinylated sohLDLR and captured LDLR with magnetic streptavidin beads, the bound CCHFV virions were detected by RT-qPCR. As shown in Fig. 4a, the biotinylated LDLR could pull-down the CCHFV virions. In addition, we also performed ELISA-based binding assays using immobilized anti-Gc mAb, anti-Gn mAb or their respective control IgGs to capture CCHFV, followed by incubation with increasing concentrations of biotinylated sohLDLR, and detection with horseradish peroxide conjugated avidin. As shown in Fig. 4b, soluble biotinylated LDLR was pulled down by CCHFV virions captured onto the ELISA plate, as indicated by increased absorbance at OD_450_. Since Gc is responsible for CCHFV entry,^29^ we then examined whether LDLR could directly interact with Gc. In vitro pull-down assays indicated that recombinant sohLDLR directly bound to the monomeric form of Gc ectodomain of both CCHFV YL16070 and IbAr 10200 strains, but not Gn (Fig. 4c; Supplementary information, Fig. S3b). Octet biolayer interferometry indicated that recombinant sohLDLR could effectively bind to the monomeric Gc ectodomain with *K*_D_ = 32.6 nM for Gc of the YL16070 strain and *K*_D_ = 42.6 nM for Gc of the IbAr 10200 strain, which is comparable to the binding between sohLDLR and VSV G protein (*K*_D_ = 54.3 nM). The binding of sohLDLR with Gc was dependent on Ca^2+^ since EDTA abolished their binding (Fig. 4d), which is consistent with the notion that LDLR binds to its physiological ligand (LDL) in a Ca^2+^-dependent manner.^30^ In these experiments, sohLDLR could not bind to Gn (Fig. 4d). Taken together, these results suggest that LDLR binds directly to Gc of CCHFV.

### LDLR is important for CCHFV pathogenesis in mice

**Fig. 5:**
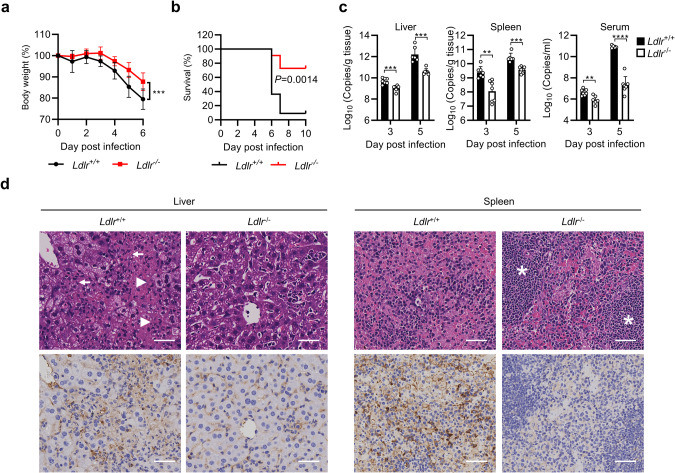
LDLR is required for CCHFV pathogenesis in mice. **a**, **b** WT (*n* = 11) and LDLR-knockout (*n* = 11) mice were pretreated with anti-IFNAR1 monoclonal antibody MAR1-5A3 (200 µg/mouse) 24 h before infection and then infected with CCHFV (10 TCID_50_) via the intraperitoneal route. Forty-eight hours post infection, 200 µg of MAR1-5A3 was administrated. The body weight (**a**) and survival (**b**) of the mice were monitored daily. **c**, **d** WT (*n* = 7) and LDLR-knockout (*n* = 7) mice were pretreated with anti-IFNAR1 monoclonal antibody MAR1-5A3 (200 µg/mouse) 24 h before infection and then infected with CCHFV (10 TCID_50_) via the intraperitoneal route. Forty-eight hours post infection, 200 µg of MAR1-5A3 was administrated. Mice were necropsied and the livers and spleens were collected at day 3 and 5 post infection. Viral loads were quantified by RT-qPCR and shown as the number of viral RNA copies per microgram of organs or per mL of sera (**c**). H&E staining and immunostaining with anti-Gn mAb (7A11) were performed and the pathological changes were indicated (**d**). The extensive necrosis (white arrowheads) and necrotic cellular debris (white arrows) in the liver were indicated; white pulps in the spleen (white asterisks) were marked. The bars represent 100 µm. Data are represented as mean ± SD. **P < 0.01; ***P < 0.001; ****P < 0.0001.

To assess the physiological importance of LDLR as a CCHFV entry receptor in vivo, we utilized LDLR-deficient mice. Since immune competent mice are resistant to CCHFV infection, we used an anti-IFNAR1 monoclonal antibody (MAR1-5A3) to transiently suppress type I IFN-triggered antiviral effects in CCHFV infection as described previously.^31^ Eight-week-old female WT and *Ldlr*^*−/−*^ mice were administrated with MAR1-5A3 for 24 h, and then challenged with CCHFV (10 TCID_50_ per mouse intraperitoneally) followed by daily monitoring. As shown in Fig. 5a, b, LDLR-deficient mice exhibited significantly less body weight loss and higher survival rate. Since CCHFV infection results in high viral loads and significant pathologic changes in the liver and spleen in type I IFN signaling-impaired mice,^31–35^ we collected the liver, spleen as well as sera of WT and LDLR knockout mice for analyses of viral loads or histopathology. As shown in Fig. 5c, viral loads in the liver, spleen and sera were significantly reduced in the LDLR knockout mice (Fig. 5c). Histopathological analyses revealed that the livers of WT mice infected with CCHFV exhibited massive necrosis and congestion, whereas LDLR knockout mice had much lower degree of liver injury. The spleens of the WT mice infected with CCHFV showed extensive necrosis and the boundary between the white pulp and red pulp was ambiguous, whereas the spleens of LDLR knockout mice had clearly visible boundary between the white and red pulp and much reduced necrosis (Fig. 5d). Immunohistochemical analyses showed dramatically reduced CCHFV Gn-positive cells in the liver and spleen of the LDLR-deficient mice (Fig. 5d).

**Fig. 6:**
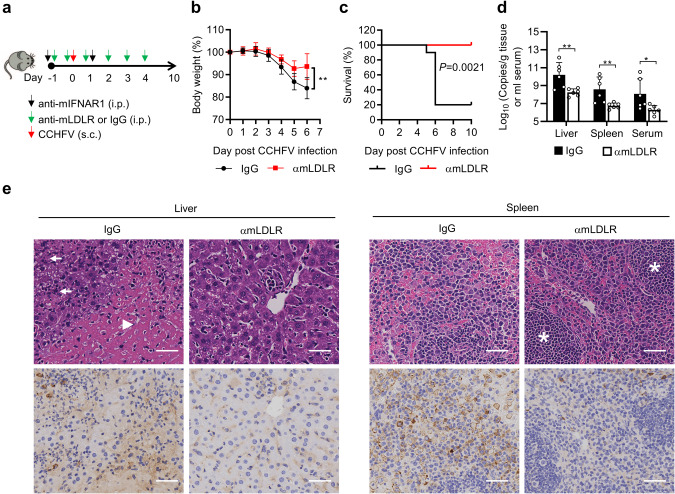
Anti-LDLR treatment protects mice from CCHFV pathogenesis in mice. **a** A flowchart of the experiment. C57BL/6 mice were pretreated with anti-IFNAR1 monoclonal antibody MAR1-5A3 (300 µg/mouse) plus control rIgG (*n* = 10, 100 µg/mouse) or an LDLR blocking antibody (R004) (*n* = 7, 100 µg/mouse) intraperitoneally 24 h before infection. Another dose of the rIgG or R004 mAb (100 µg) was administrated 1 h prior to infection. Mice were challenged with CCHFV (100 TCID_50_ per mouse, subcutaneously). Twenty-four hours post infection, MAR1-5A3 (200 µg/mouse) was administrated. The rIgG or R004 mAb was administrated at 100 µg quaque die for 5 days post infection. **b**, **c**, Protective effects of LDLR blocking antibody on fatality caused by CCHFV infection. As described in (**a**), C57BL/6 mice were treated with rIgG (*n* = 10) or the LDLR blocking antibody (R004) (*n* = 7) and subjected to CCHFV challenge. Body weight (**b**) and survival (**c**) of the mice were monitored daily. **d**, **e** Protective effects of LDLR blocking antibody on CCHFV caused pathogenesis. As described in (**a**), C57BL/6 mice were treated with rIgG (*n* = 6) or the LDLR blocking antibody (R004) (*n* = 6) and subjected to CCHFV challenge. Mice were necropsied and the livers and spleens were collected at day 5 post infection. Viral loads were quantified by qRT-PCR and shown as the number of viral RNA copies per microgram of organs or per mL of sera (**d**). H&E staining and immunostaining with anti-Gn mAb (7A11) were performed and the pathological changes were indicated (**e**). The extensive necrosis (white arrowheads) and necrotic cellular debris (white arrows) in liver were indicated; white pulps in the spleen (white asterisks) were marked. The bars represent 100 µm. Data are represented as mean ± SD. *P < 0.05; **P < 0.01.

We next evaluated whether the LDLR blocking antibody would impair CCHFV infection in C57BL/6 mice. Wild-type C57BL/6 mice were pretreated with the anti-IFNAR1 antibody (MAR1-5A3) plus the anti-mLDLR antibody (R004) or control IgG intraperitoneally 24 h before infection. To avoid potential influence on viral infection by antibodies inoculated via the same route, mice were challenged with CCHFV via subcutaneous route in this experiment. After CCHFV infection (100 TCID_50_ per mouse, a dose at which fatality could be caused via subcutaneous route), the mice were administrated with the anti-mLDLR antibody (R004) or control IgG daily for 5 days (Fig. 6a). As shown in Fig. 6b, c, treatment with the anti-mLDLR antibody significantly reduced body weight loss and lethality in CCHFV-infected mice. Consistently, viral loads (Fig. 6d), pathological injury and CCHFV Gn-positive cells (Fig. 6e) were dramatically reduced in the liver and spleen of mice treated with the anti-mLDLR antibody. These results suggest that LDLR plays an important role in CCHFV infection and pathogenesis in mice.

## Discussion

CCHFV was isolated in 1956 and recognized as the pathogen of CCHF in 1969. Unfortunately, a bona-fide entry receptor for CCHFV has not been identified until the current study, which has hindered the understanding of CCHFV–host interaction as well as development of efficient treatment for CCHFV infection. Although it has been reported that the nucleolar protein nucleolin can interact with CCHFV glycoprotein Gc,^29^ a role of nucleolin in CCHFV entry was not established. Another study has proposed that the C-type lectin receptor, DC-SIGN, is a potential entry factor for CCHFV.^36^ However, DC-SIGN is exclusively expressed on dendritic cells and macrophages, and binds to the glycans presented on viral glycoproteins non-specifically. In addition, anti-DC-SIGN treatment only partially inhibits CCHFV infection.^36^ Considering the broad cell and tissue tropisms of CCHFV,^37^ there must exist an ubiquitously expressed and potent entry receptor for CCHFV.

Several lines of evidence in our study suggest that LDLR is a general entry receptor for CCHFV infection from mouse to human. Firstly, the surface levels of LDLR are positively correlated with the infectivity of CCHFV in distinct cell types. Second, knockout of LDLR impairs CCHFV infection in diverse cell types from mouse to human. Third, LDLR blocking antibodies or soluble LDLR-Fc fusion protein dose-dependently impair CCHFV infection in various cell types. Fourth, knockout of LDLR in susceptible cells impairs CCHFV binding to the cells and its internalization, whereas knockout of the LDLR adapter protein LDLRAP1, which is important for LDLR-mediated endocytosis,^26^ impairs the internalization of CCHFV but not its binding to the cells. Fifth, mutagenesis and reconstitution experiments demonstrate that the LBD in the extracellular fragment of LDLR is required for CCHFV infection. In vitro biochemical experiments indicate that CCHFV Gc but not Gn directly interacts with sohLDLR with high affinity in a Ca^2+^-dependent manner. The affinity between sohLDLR and CCHFV Gc is comparable to that of VSV G.^23^ Finally, genetic knockout of LDLR or administration of an LDLR blocking antibody significantly reduces CCHFV infection and pathogenesis in mice. Collectively, these results establish LDLR as a general entry receptor for CCHFV infection of a broad range of cell types from mouse to human. LDLR is conserved across different species, making it an ideal receptor for CCHFV transmission from intermediate animal hosts, such as cattle, sheep, goats, camels, hares, and mice, to humans. In addition, LDLR is expressed in almost all human tissues, predominantly in liver, lung, placenta, kidney, spleen, and brain,^38^ which is correlated with the broad tissue tropism of CCHFV in vivo, as CCHFV is most likely to be found in the liver, kidney, lung, spleen, bone marrow and brain at autopsy.^39^

Interestingly, our study suggests that LDLR is not utilized for entry by other examined bunyaviruses including RVFV and EBIV. LDLR-deficiency or treatment with sohLDLR or an LDLR blocking antibody has no marked effects on infection of RVFV or EBIV in SW13 cells. These results suggest that LDLR is specifically utilized by CCHFV for entry but not generally utilized by bunyaviruses.

In our experiments, we found that knockout of LDLR or treatment with sohLDLR abolished CCHFV infection in human SW13 cells. However, LDLR-deficiency or sohLDLR treatment in other examined cell types greatly reduced but did not abolished CCHFV infectivity. In addition, CCHFV infectivity was dramatically reduced but not abolished in primary mouse hepatocytes and lung fibroblasts derived from LDLR knockout mice. Pre-treatment with an anti-human LDLR monoclonal antibody dose-dependently reduced CCHFV infection in primary HUVECs while had limited inhibition in primary human PBMCs. Furthermore, CCHFV infection and pathogenesis was reduced but not blocked in LDLR knockout mice, or wild-type mice administrated with LDLR blocking antibody. These observations suggest that additional entry receptor(s) or coreceptor(s) as well as different entry route may exist for CCHFV in certain cell types.

In our study, we noticed that whereas treatment with the LDLR blocking antibody conferred 100% protection against CCHFV caused fatality in mice, LDLR-knockout only ends up with partial protection. It has been well demonstrated that LDLR-deficient mice have dyslipidermia,^40^ which may exacerbate inflammatory responses flowing microbial infections.^41–44^ Therefore, we speculate that the hyperlipidermia of *Ldlr*^*−/−*^ mice renders them more vulnerable to the fatal outcomes of CCHFV infection. Previous studies have demonstrated that LDLR is also a receptor for VSV,^23^ Hepatitis C Virus (HCV),^45^ and human rhinovirus 2 (HRV2).^46^ However, LDLR-deficiency did not abolish VSV,^23^ HCV^47,48^ and HRV2 infection,^46^ suggesting that LDLR is redundant for entry of these viruses. In fact, alternative receptors have been suggested for VSV, HCV and HRV2.^23,46,49–52^ In our experiments, we found that sohLDLR-Fc impaired both CCHFV and VSV infection in SW13 cells, however, the anti-human LDLR mAb R301 only blocked CCHFV but not VSV infection in these cells. The simplest explanation for these observations is that CCHFV and VSV bind different regions of LDLR for cell entry and the R301 mAb binds to an epitope of LDLR that is required for binding of CCHFV but not VSV.

Recently, certain LRP family members have been identified as receptors for some viruses. For example, LDLRAD3 has been shown as a receptor for Venezuelan Equine Encephalitis Virus (VEEV),^53^ VLDLR and ApoER2 as receptors for SFV, Eastern Equine Encephalitis Virus (EEEV) and Sindbis Virus (SINV),^28^ and LRP1 as a receptor for RVFV in mice.^54^ These studies suggest that LDLR and LRPs may be evolutionarily conserved “hotspots” for pathogen interaction. Collectively, our current study has identified LDLR as an important cellular receptor for CCHFV, which would advance the understanding of CCHFV-host interaction and CCHFV pathogenesis. In this study, we also demonstrate that LDLR-deficiency, LDLR blocking antibodies or soluble LDLR proteins can impair CCHFV infection in cells and mice. Therefore, targeting CCHFV–LDLR interaction such as by anti-LDLR antibody, soluble LDLR or small molecules inhibiting LDLR ligand binding or its internalization may provide effective therapeutic strategies for the treatment of CCHFV infectious diseases.

## Materials and methods

### Mice

Wild type and LDLR-knockout (T001464) C57BL/6J mice were purchased from Gempharmatech Co., Ltd. Mice were group-housed with 12-h dark/light cycle and had access to food and water *ad libitum*. All animal experiments were performed in compliance with the policies of Wuhan Institute of Virology, Chinese Academy of Sciences, and were approved by the Institutional Animal Care and Use Committee (IACUC) (Approval Number: WIVA31202301).

### Cells

SW13, HEK293T, Vero E6 (all from ATCC), Huh7, HUVEC (provided by National Virus Resource Center, Wuhan, China) and DLD1 (provided by Dr. Youjun Li, Wuhan University) cells were cultured at 37 °C in Dulbecco’s modified Eagle’s medium (DMEM) (GIBCO) supplemented with 10% (v/v) fetal bovine serum (FBS) and 1% (v/v) penicillin-streptomycin (Hyclone). The primary mouse hepatocytes and MLF cells were isolated and cultured as previously described.^55,56^ Primary human PBMCs were isolated from whole blood of healthy donors with SepMate™-15 (STEMCELL Technologies) according to the manufacturer’s instructions and cultured in RPMI 1640 medium supplemented with 10% (v/v) fetal bovine serum (FBS) and 1% (v/v) penicillin-streptomycin. The isolation of human PBMCs and its application for virus infection were approved by Institutional Review Board, Wuhan Institute of Virology, Chinese Academy of Sciences (Approval Number: WIVH31202301). FreeStyle 293F cells (provided by Dr. Bing Yan, Wuhan Institute of Virology, Chinese Academy of Sciences) were cultured at 37 °C in SMM 293-TII Expression Medium (SinoBiological) on an orbital shaker platform rotating at 120 rpm. All cell lines were tested and found to be free of mycoplasma contamination using the MycoBlue Mycoplasma Detector kit (#D101, Vazyme).

### Viruses

The CCHFV (YL16070 strain, GenBank accession number: KY354082)^57^ used in this study was provided by the National Virus Resource Center (Wuhan, China). The NSs-deficient RVFV (RVFV-r∆NSs-eGFP, BJ01 strain) and EBIV (Cu-XJ20 isolate) were provided by Dr. Ke Peng and Dr. Han Xia, respectively. CCHFV was propagated in Vero E6 cells and TCID_50_ was determined in SW13 cells. RVFV-r∆NSs-eGFP and EBIV were propagated as previously described.^58,59^ All experiments with live CCHFV were performed in the BSL-3 facilities of Wuhan Institute of Virology, Chinese Academy of Sciences.

### Antibodies

Mouse anti-Flag monoclonal antibody (#F3165-M2, Sigma-Aldrich), rabbit anti-β-actin monoclonal antibody (#AC026, ABclonal), rabbit anti-hLDLR monoclonal antibody (#10231-R301, SinoBiological), rabbit anti-hLDLR monoclonal antibody (#A14996, ABclonal), rabbit anti-mLDLR monoclonal antibody (#50305-R004, SinoBiological), mouse anti-IFNAR1 monoclonal antibody (MAR1-5A3, #BE0241, BioXCell), mouse anti-Gn monoclonal antibody (JE12, #MAB12317, The Native Antigen Company), phycoerythrin (PE)-conjugated anti-hLDLR monoclonal antibody (#10231-R301-P, SinoBiological), Alexa Fluor™ 488-conjugated anti-mouse IgG (#R37114, Thermo Fisher), human control IgG (#12000C, Invitrogen), mouse control IgG (#I5381, Sigma-Aldrich), rabbit control IgG (#CR1, SinoBiological) and goat control IgG (#CR2, SinoBiological) were purchased from the indicated companies. Human anti-Gc monoclonal antibody ADI-36121 was prepared as previously reported^15^ and provided by Dr. Bing Yan at Wuhan Institute of Virology, Chinese Academy of Sciences. Mouse anti-Gn monoclonal antibody (clone 7A11) was customized from ABclonal and further validated by us.

### CRISPR/Cas9 knockout

Gene editing was performed with the CRISPR/Cas9 system. Briefly, double-stranded oligonucleotides corresponding to the target sequences were cloned into the lenti-CRISPR-V2 vector, which was co-transfected with the packaging plasmids psPAX2 and pMD2.G into HEK293T cells. Two days after transfection, lentiviruses were harvested and used to infect target cells. The infected cells were selected with puromycin (1 μg/mL) for at least 7 days. The sequences of gRNAs are shown in Supplementary information, Table S1. The LDLR-deficient clone was obtained by limited dilution. *LDLR* gene-mutation and its deficiency were confirmed by Sanger sequencing and immunoblots respectively.

### RT-qPCR

Total RNA from the cells was isolated with RNAiso Plus (#9109, TaKaRa) and reverse transcription of 1 μg of RNA was conducted with the cDNA synthesis kit (#R212, Vazyme) according to the manufacturer’s instructions. For extraction of viral RNA in cell culture supernatant, TaKaRa MiniBEST Viral RNA/DNA Extraction Kit Ver.5.0 was used (#9766, TaKaRa). Quantitative PCR was performed as previously described.^60^ The threshold cycle (Ct) for the indicated genes was normalized to that of the housekeeping gene *GAPDH* and shown as the relative mRNA level. Gene-specific primers used in this study are listed in Supplementary information, Table S2.

### RNA interference

The siRNA duplexes targeting the indicated LRP family members were chemically synthesized by RiboBio. Fifty nanomolar siRNA duplexes were transfected into Huh7 cells using the PepMute siRNA transfection reagent (SignaGen Laboratories) according to the manufacturer’s instructions. Sixty hours after transfection, the medium was replaced with fresh medium and cells were further incubated for 24 h. The sequences of siRNA oligonucleotides are included in Supplementary information, Table S3.

### Flow cytometry

Cells were seeded on six-well plates overnight and scraped off the plate and washed with PBS. For detection of surface expression of LDLR, cells were suspended with 200 μL PBS and incubated with phycoerythrin (PE)-conjugated anti-LDLR monoclonal antibody (#10231-R301-P, Sino Biological) at 2 µg/mL on ice for 1 h, and then fixed with 4% paraformaldehyde for 15 min. Cells were then washed with PBS and analyzed by flow cytometry.

For intracellular detection of CCHFV Gn, cells were fixed with 4% paraformaldehyde for 15 min, permeabilized with Perm/Wash Buffer (#554723, BD) for 15 min and then stained with the anti-Gn monoclonal antibody (7A11, customized from ABclonal) for 1 h. After washing with Perm/Wash Buffer for 3 times, cells were stained with Alexa Fluor™ 488-conjugated anti-mouse IgG for 30 min. After staining, cells were washed PBS and analyzed by flow cytometry.

### Crystal violet staining

Control and *LDLR*-edited SW13 cells were infected with CCHFV at the indicated MOI for 72 h. Cells were then fixed with 4% paraformaldehyde for 15 min and stained with 1% crystal violet for 30 min before photographing.

### TCID_50_ assay

Cells were plated in 96-well plates overnight before subjected to TCID_50_ assays. Briefly, CCHFV stock was serially diluted from 1:10 to 1:10^6^ with DMEM. SW13 cells were incubated with 100 μL of each diluted stock for 1 h. The viruses were then removed and 150 μL of DMEM containing 2% FBS was added to cells to maintain cell growth for 5 days. The cytopathic effects were observed and viral titers were calculated using the Reed–Muench method.^61^

### Reconstitution of LDLR-deficient cells

cDNAs encoding a C-terminal Flag-tagged full-length human LDLR (NM_000527.5) or its truncations lacking the LBD (aa24–313) or EFG-like domain (aa314–712) were cloned into the retrovirus vector pMSCV. The sgRNA targeting sequence of LDLR was mutated synonymously in these plasmids to avoid editing of the reconstituted cDNA by Cas9.

Reconstitution of LDLR or its mutants into LDLR-deficient cells was performed by retrovirus-mediated transduction. Briefly, HEK293T cells plated on 100 mm dishes were transfected with the retroviral plasmid (10 μg) together with pGag-pol (10 μg) and pVSV-G (3 μg). Two days after transfection, the retroviruses were harvested and used to infect LDLR-deficient SW13 cells in the presence of polybrene (8 μg/mL). The infected cells were selected with blastcidin (1 μg/mL) for at least 7 days. Reconstituted cells were assessed for expression of LDLR or its truncations by immunoblots using a rabbit polyclonal antibody against LDLR (#A14996, ABclonal).

### Assays for viral attachment and internalization

For viral attachment assay, WT and LDLR-deficient SW13 cells were seeded on 12-well plates overnight. The cells were incubated with CCHFV (MOI = 5) on ice for 1 h. After 5 times of washing with ice-cold PBS, cells were collected and RNAs were extracted for RT-qPCR analysis. For the internalization assay, following the on-ice incubation and washing, the cells were then incubated at 37 °C for 1 h. Cells were then washed once with PBS and treated with 500 ng/mL proteinase K for 1 h on ice to stop endocytosis and degrade viruses that have not been internalized. The cells were then washed 3 times with PBS and collected for RT-qPCR analysis.

### Blocking assays with anti-LDLR antibodies, soluble LDLR-Fc fusion protein or LDL

Cells were seeded on 12-well plates overnight. For antibody blocking, cells were pre-incubated with the indicated concentrations of anti-hLDLR (#10231-R301, SinoBiological), anti-mLDLR (#50305-R004, SinoBiological) or control IgG (#CR1, SinoBiological) for 1 h at 37 °C before infection with CCHFV (MOI = 0.05), RVFV (MOI = 0.1), EBIV (MOI = 0.5) or VSV (MOI = 0.1). Twenty-four hours post infection, cells were collected for RT-qPCR of CCHFV *S* segment, RVFV *M* segment, EBIV *S* segment and VSV *L* respectively.

For blocking with the soluble LDLR protein, CCHFV (MOI = 0.05), RVFV (MOI = 0.1), EBIV (MOI = 0.5) or VSV (MOI = 0.1) were pre-incubated with the indicated concentrations of sohLDLR-Fc (#10231-H05H, SinoBiological) or the control Fc (#10690-MNAH, SinoBiological) in a volume of 100 μL for 1 h at 37 °C before inoculated to cells. Twenty-four hours post infection, cells were collected for RT-qPCR of CCHFV *S* segment, RVFV *M* segment, EBIV *S* segment and VSV *L*, respectively.

For blocking with LDL, cells were pre-treated with the indicated concentrations of LDL for 1 h and then left uninfected or infected with CCHFV (MOI = 0.05). Twenty-four hours post infection, cells were collected for RT-qPCR of CCHFV *S* segment.

### Preparation of CCHFV Gc and VSV-G proteins

The pCAGGS-GPC_10200_ (provided by Dr. Zhihong Hu), a codon optimized expression plasmid for the GPC of CCHFV IbAr 10200 strain, was used as a template for construction of the Gc expression plasmids. Briefly, a pCAGGS-based expression plasmid for Gc of the CCHFV IbAr 10200 strain (pCAGGS-Gc_10200_) was constructed by inserting a furin cleavage site (RSKR) to the 3′ of GPC_10200_ aa1–515, followed with GPC_10200_ aa1041–1579 and a 6× His tag at the C-terminus.^13^ For expression of Gc of the CCHFV YL16070 strain, the coding sequence of GPC_10200_ aa1041–1579 was replaced by that encoding the GPC aa1054–1592 of the YL16070 strain. For expression of VSV G protein, a cDNA encoding G of VSV (Indiana strain) was fused with 6× His tag and cloned into the pCAGGS vector.

FreeStyle 293F cells were seeded at 5 × 10^5^ cells/mL one day before transfection. Two hundred micrograms of the indicated expression plasmid were diluted with Opti-MEM (Thermo Fisher), complexed with PEI MAX^®^ transfection reagent (Polysciences, Inc.) following the manufacturer instructions and added to cells. For purification of CCHFV Gc, 4 days after transfection, the supernatant was collected, centrifuged at 3000× *g* for 15 min, and purified with Ni-NTA Sepharose (GE) chromatography. The eluted proteins were subsequently purified by size exclusion chromatography (SEC) using a Superdex 200 10/30 column (GE) to confirm the monomeric form of Gc. For purification of VSV-G, 4 days after transfection, cells were collected and sonicated with PBS. The cell lysate was centrifuged at 14,000× *g* for 15 min and subjected to Ni-NTA Sepharose chromatography. The purified protein was dialyzed with PBS, filtered through a 0.20-μm filter, and stored at –80 °C. The purity of each protein was confirmed by SDS PAGE and Coomassie blue staining.

### Pull-down assay

Two micrograms of biotinylated LDLR (#10231-H08H-B, SinoBiological) were mixed with CCHFV viral stock (500 μL) or CCHFV Gc protein (2 μg) in 1 mL PBS in the presence of 0.5 mM CaCl_2_ at 4 °C for 2 h. Then 30 μL of streptavidin magnetic beads (#HY-K0208, MedChemExpress) were added and mixed by rotating at 4 °C for 30 min. The magnetic beads were collected and washed three times with PBS containing 0.5 mM CaCl_2_. For RT-qPCR experiments, the beads were subjected to RNA extraction with 500 μL RNAiso Plus (#9109, TaKaRa). For immunoblots, the beads were treated with 80 μL 2× SDS loading buffer and subjected to SDS-PAGE.

### ELISA-based binding assay

The binding of sohLDLR to CCHFV virions was determined by ELISA as previously described^62^ with modifications. Monoclonal antibodies against CCHFV Gc (ADI 36121, prepared as previously reported^15^ and provided by Dr. Bing Yan at Wuhan Institute of Virology), Gn (JE12, #MAB12317, The Native Antigen Company) or the respective control IgG (#12000C, Invitrogen; #I5381, Sigma-Aldrich) (50 μL, 0.5 μg/mL) were immobilized on Maxisorp ELISA plates (Thermo Fisher) overnight at 4 °C in PBS. The plates were washed 4 times with PBS and blocked with PBS supplemented with 4% BSA for 1 h at room temperature. CCHFV virus stock was then added (50 μL/well) to the plates and incubated for 1 h at room temperature, followed by 3 times washing with PBS. Biotinylated LDLR (#10231-H08H-B, SinoBiological) diluted in PBS supplemented with 2% BSA was then added at the indicated concentrations and incubated for 1 h at room temperature. After 5 times washing with PBS, the plates were incubated with Avidin-HRP (1:1000 dilution, #405103, Biolegend) for 1 h at room temperature, followed by 5 times washing with PBS. Finally, TMB (3,3′-5,5′ tetramethylbenzidine) was added, and 2 N H_2_SO_4_ was applied to stop the reaction. Absorbance was read at 450 nm with the microplate reader (Biotech).

### ForteBio Octet Red Bio-layer interferometry

The binding affinities between sohLDLR and Gc of the CCHFV YL16070 strain, Gc or Gn (#REC31615, The Native Antigen Company) of the CCHFV IbAr 10200 strain, were measured by the ForteBio Octet Red system (ForteBio, Inc). The biotinylated sohLDLR (25 μg/mL) was immobilized onto the Streptavidin biosensors, the association and dissociation of the indicated protein to sohLDLR were monitored in 200 μL of PBS containing 0.02% Tween 20, 0.5 mM CaCl_2_ and 1 mg/mL BSA. The dissociation constants were calculated with the Octet RED software.

### CCHFV infection of mice

1. For evaluation of LDLR-deficiency on CCHFV infection, 8-week-old female WT and LDLR-deficient mice were anesthetized with isoflurane and administrated with anti-IFNAR1 monoclonal antibody MAR1-5A3 (#BE0241, BioXCell) (200 µg/mouse) 24 h prior to viral infection. CCHFV was inoculated intraperitoneally (10 TCID_50_/mouse) and 200 µg of anti-IFNAR1 monoclonal antibody was administrated 48 h post infection.
2. For assessment of a protective role of LDLR blocking antibody, 8-week-old female C57BL/6 J mice were anesthetized with isoflurane and administrated with 300 µg anti-IFNAR1 monoclonal antibody MAR1-5A3 (#BE0241, BioXCell) plus 100 µg LDLR blocking antibody (#50305-R004, SinoBiological) or control IgG (#CR1, SinoBiological) intraperitoneally 24 h prior to challenge. CCHFV was inoculated subcutaneously (100 TCID_50_/mouse) and anti-IFNAR1 monoclonal antibody (200 µg/mouse) was administrated 24 h post infection. The anti-LDLR or the control IgG was administrated intraperitoneally at 100 µg quaque die for 5 days post infection.

For both (1) and (2), following CCHFV challenge, mice were monitored daily for body weight. Mice were euthanized when exhibited a weight loss of more than 20%, crawling difficulty, and/or showed no response to touch stimulation. A subset of mice were euthanized at 5 dpi, and livers and spleens were collected for further measurement of viral loads and pathological analysis. Tissue RNA was extracted and RT-qPCR was performed to quantify the amount of vRNA in each tissue as previously described.^63^

### Statistics

Statistical analysis was performed with Prism Version 8.0 (GraphPad). Statistical significance was analyzed by two-way ANOVA analysis, followed by Dunnett’s test. Two-tailed unpaired (Student’s) *t*-test was performed if only two conditions were compared. For the animal survival study, Kaplan–Meier survival curves were generated and analyzed by Log-Rank test. Statistical significance was assigned when *P* values were < 0.05. Error bars show mean and standard deviation (mean ± SD) unless otherwise specified. All data are representative of at least two independent experiments with similar results.

### Supplementary information

Supplementary information, Fig. S1

Supplementary information, Fig. S2

Supplementary information, Fig. S3

Supplementary information, Table S1

Supplementary information, Table S2

Supplementary information, Table S3

## Data Availability

All the data supporting the findings of this study are available within the article and its supplementary information files, or can be obtained from the corresponding author upon reasonable request.
