# Supplementary material for: LDLR is an entry receptor for Crimean-Congo hemorrhagic fever virus
Source: Cell Res. 2024 Jan 5;34(2):140–50. doi: 10.1038/s41422-023-00917-w (PMC10837205; doi:10.1038/s41422-023-00917-w)
Supplement: Supplementary file 1 — Supplementary information, Fig. S1 [file 41422_2023_917_MOESM1_ESM.pdf]

a

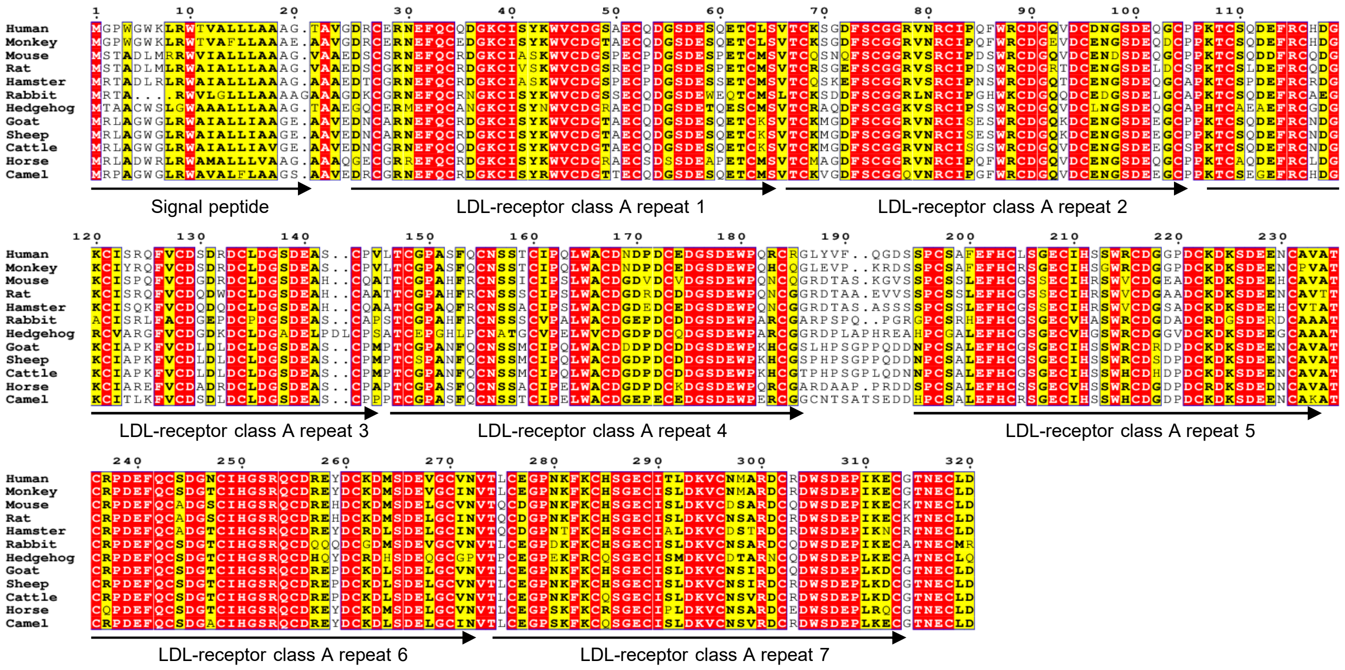

b

	Human	Monkey	Mouse	Rat	Hamster	Rabbit	Hedgehog	Goat	Sheep	Cattle	Horse	Camel
Human		93.4	77.3	76.3	74.8	72.6	68.2	80.8	80.8	80.5	80.1	81.7
Monkey	96.3		75.1	75.7	73.8	70.7	67	81.4	81.1	80.5	79.1	81.7
Mouse	84.4	82.9		89.4	86.3	71.4	66.7	74.5	74.2	74	74.8	72.7
Rat	83.5	82.2	92.8		86	71.4	66	74.5	74.8	74.8	74.8	74.2
Hamster	81.9	81.9	91.3	92.8		69.9	67	74.5	74.8	74.5	74.8	74.8
Rabbit	82.2	80.9	81.6	82.6	81.3		66.7	71.8	71.5	71.2	72.4	70.3
Hedgehog	79.9	78.4	77.8	77.5	78.7	78.1		67.6	67.6	67.6	71	68.5
Goat	88.5	88.2	83.5	82.6	82.3	82	77.5		98.4	95	80.1	83.5
Sheep	87.6	87.3	83.5	82.6	82.3	82	77.8	98.8		95.7	80.1	83.9
Cattle	87.3	87.6	83.9	83.5	83.2	82	77.8	96.9	97.2		79.2	84.8
Horse	87.5	86.9	83.8	83.5	84.1	81.9	80.9	87.6	87.3	86.6		79.5
Camel	88.8	87.6	83.2	83.5	83.5	82.3	78.1	90.7	90.7	91.6	87.9	

Identity

Similarity

Supplementary information, Fig. S1. Sequence alignment of LBD of LDLR across different species. a, Sequence alignment of LBD of human LDLR (*Homo sapiens*, NP_000518.1) to its orthologues from monkey (*Macaca mulatta*, XP_014978386.2), mouse (*Mus musculus*, NP_034830.2), rat (*Rattus norvegicus*, NP_786938.2), hamster (*Mesocricetus auratus*, XP_005078593.1), rabbit (*Oryctolagus cuniculus*, NP_001265794.1), hedgehog (*Erinaceus europaeus*, XP_060037767.1), goat (*Capra hircus*, XP_005682432.1), sheep (*Ovis aries*, XP_004008580.2), cattle (*Bos taurus*, NP_001160002.1), horse (*Equus caballus*, XP_005611994.2) and camel (*Camelus dromedarius*, XP_010976615.1) using ESPript 3. Red and yellow boxes indicate conserved and similar residues between orthologues, respectively. The LDL-receptor class A repeats are indicated below the sequence. **b,** The identity (green) and similarity (yellow) matrix of LBD of LDLR across species. The matrix was generated using MatGat 2.01.
