# Supplementary material for: LDLR is an entry receptor for Crimean-Congo hemorrhagic fever virus
Source: Cell Res. 2024 Jan 5;34(2):140–50. doi: 10.1038/s41422-023-00917-w (PMC10837205; doi:10.1038/s41422-023-00917-w)
Supplement: Supplementary file 2 — Supplementary information, Fig. S2 [file 41422_2023_917_MOESM2_ESM.pdf]

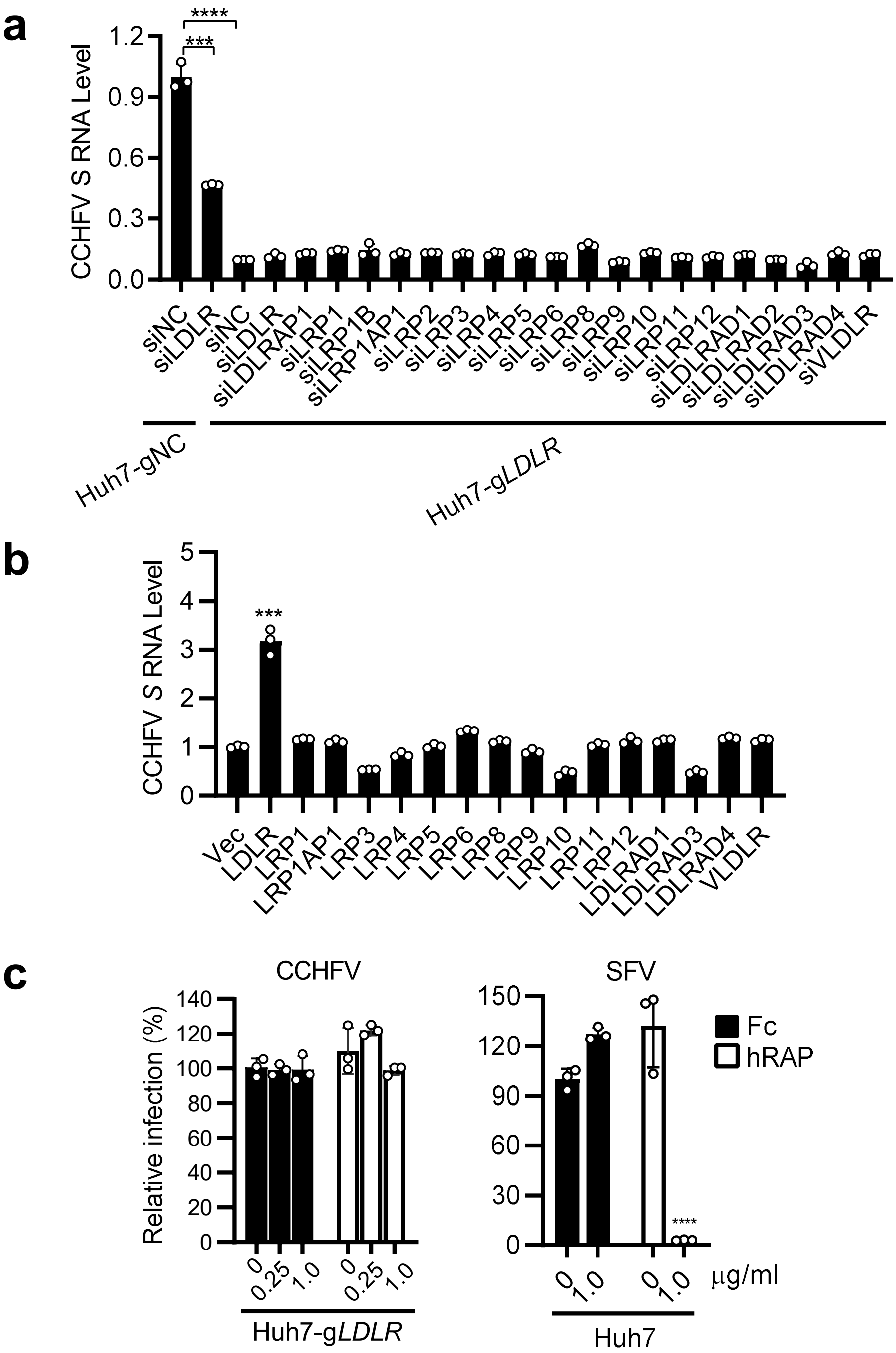

Supplementary information, Fig. S2. Effects of LRPs on CCHFV infection. a, Effects of knockdown of LRPs on CCHFV infection in LDLR-deficient Huh7 cells. LDLR-deficient or control Huh7 cells were transfected with siRNAs targeting LDLR or the indicated LRP family members for 48 h and then were infected with CCHFV (MOI=0.05) for 24 hours before RT-qPCR was performed to measure CCHFV S mRNA levels. **b,** Effects of ectopic expression of LDLR and LRPs on CCHFV infection in DLD1 cells. DLD1 cells were transiently transfected with LDLR and the indicated LRP family members for 24 h and then were infected with CCHFV (MOI=0.05) for 24 h before RT-qPCR was performed to measure CCHFV S mRNA levels. **c,** Effects of soluble human RAP protein on CCHFV and SFV infection. LDLR-deficient or control Huh7 cells were pre-incubated with RAP or Fc for 1 h at the indicated concentrations before CCHFV or SFV infection. Twenty-four hours after infection, cells were collected for RT-qPCR analysis of CCHFV S mRNA or SFV NSP1 mRNA levels. The relative infectivity was calculated by normalization to the mRNA level in cells without Fc treatment.
