# Supplementary material for: LDLR is an entry receptor for Crimean-Congo hemorrhagic fever virus
Source: Cell Res. 2024 Jan 5;34(2):140–50. doi: 10.1038/s41422-023-00917-w (PMC10837205; doi:10.1038/s41422-023-00917-w)
Supplement: Supplementary file 3 — Supplementary information, Fig. S3 [file 41422_2023_917_MOESM3_ESM.pdf]

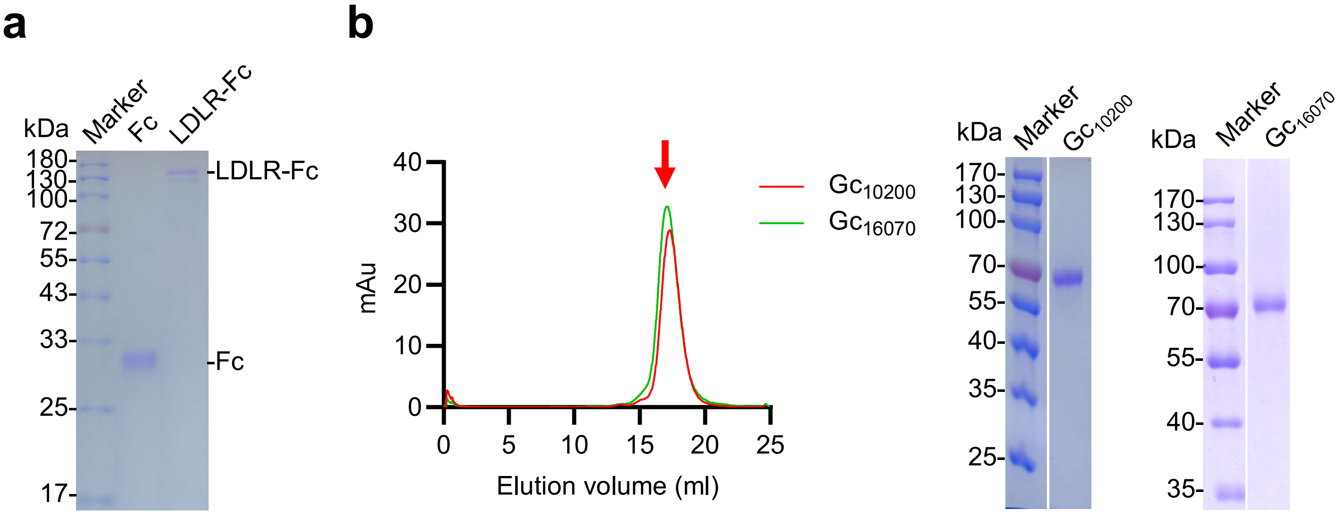


**Supplementary information, Fig. S3. Characterization of soluble LDLR and CCHFV Gc proteins. a,** Validation of Fc and LDLR-Fc. Commercially purchased Fc (#10690-MNAH,SinoBiological) and soluble LDLR-Fc (#10231-H05H, SinoBiological) were subjected to SDS-PAGE and analyzed by Coomassie staining. **b,** Purification and characterization of CCHFV Gc. Gc of CCHFV IbAr 10200 or YL16070 strain purified by Ni-NTA Sepharose chromatography was subjected to SEC with Superdex200 10/300 columns, respectively (left). The eluted fractions were then collected and subjected to SDS-PAGE and analyzed by Coomassie staining (right).
