# Supplementary material for: LDLR is an entry receptor for Crimean-Congo hemorrhagic fever virus
Source: Cell Res. 2024 Jan 5;34(2):140–50. doi: 10.1038/s41422-023-00917-w (PMC10837205; doi:10.1038/s41422-023-00917-w)
Supplement: Supplementary file 4 — Supplementary information, Table S1 [file 41422_2023_917_MOESM4_ESM.pdf]

Supplementary information, Table S1. sgRNA sequences

sgRNA	Forward sequence (5'→3')	Reverse sequence (5'→3')
Human LDLR#1	TCAAGCATCGATGTCAACGG	CCGTTGACATCGATGCTTGA
Human LDLR#2	GTCATAGGAAGAGACGCCGT	ACGGCGTCTCTTCCTATGAC
Human LDLR#3	CTTAAGGTCATTGCAGACGT	ACGTCTGCAATGACCTTAAG
Monkey LDLR#1	GAACGTGGTCGCTCTGGACA	TGTCCAGAGCGACCACGTTC
Monkey LDLR#2	TGCACTCGCCACTTCGACAG	CTGTCGAAGTGGCGAGTGCA
Mouse Ldlr#1	ACAGTCGACATCCCCGTCGC	GCGACGGGGATGTCGACTGT
Mouse Ldlr#2	AGTCTACCGCCAGCCCGTCA	TGACGGGCTGGCGGTAGACT
Human LRP1#1	GCCCGATCCACAGACAGACG	CGTCTGTCTGTGGATCGGGC
Human LRP1#2	CGATGCGCCGGATTTCATGG	CCATGAAATCCGGCGCATCG
Human LRP1B#1	ACTCGGACATTGTGGTCGCC	GGCGACCACAATGTCCGAGT
Human LRP1B#2	TATTCACGTGATGAGCAAGT	ACTTGCTCATCACGTGAATA
Human LRP2#1	GATGAACTACCACCGACCGT	ACGGTCGGTGGTAGTTCATC
Human LRP2#2	GAACGCGGGCTATCACAGTG	CACTGTGATAGCCCGCGTTC
Human LRP3#1	CGACTACGTGCAGGTATACG	CGTATACCTGCACGTAGTCG
Human LRP3#2	GACCTGTTCGGCGCCGCTCG	CGAGCGGCGCCGAACAGGTC
Human LRP4#1	AGTCATCGTCGCCATCGCAG	CTGCGATGGCGACGATGACT
Human LRP4#2	GGACTATGATACCCGCAGGT	ACCTGCGGGTATCATAGTCC
Human LRP5#1	GTGCGATGACCAGAGCGACG	CGTCGCTCTGGTCATCGCAC
Human LRP5#2	GTTGGACGACTCGATCATGT	ACATGATCGAGTCGTCCAAC
Human LRP6#1	TCGCGTTGGACCCTGCCGAA	TTCGGCAGGGTCCAACGCGA
Human LRP6#2	GTCCACGTATGAAGGGAGAT	ATCTCCCTTCATACGTGGAC
Human LRP8#1	TCGGCGGCCACCATCAACTG	CAGTTGATGGTGGCCGCCGA
Human LRP8#2	GTGCTACCCTGGCTACGAGA	TCTCGTAGCCAGGGTAGCAC
Human LRP9#1	TCATACGGGCAAAACCTCCG	CGGAGGTTTTGCCCGTATGA
Human LRP9#2	GTAGTAATTGGGCAGACACG	CGTGTCTGCCCAATTACTAC
Human LRP10#1	GGTGCCCGCCGTCGTCAGCG	CGCTGACGACGGCGGGCACC
Human LRP10#2	GCAAGCAATAGCCCCGCACA	TGTGCGGGGCTATTGCTTGC
Human LRP11#1	GTCGCGCGCTTACCCTGCCG	CGGCAGGGTAAGCGCGCGAC
Human LRP11#2	GCGCGAACTTGCAGACGTTG	CAACGTCTGCAAGTTCGCGC

Human LRP12#1	ACTTACCGTACACCCCAGCG	CGCTGGGGTGTACGGTAAGT
Human LRP12#2	GTATGACTCAGGGGCTACGC	GCGTAGCCCCTGAGTCATAC
Human LDLRAD1#1	TGATGGCACTAACAACTGCG	CGCAGTTGTTAGTGCCATCAC
Human LDLRAD1#2	CATGAACAAGGTCTTCCCCC	GGGGGAAGACCTTGTTCATG
Human LDLRAD2#1	TTCGCCCACGAAGTCCACGC	GCGTGGACTTCGTGGGCGAA
Human LDLRAD2#2	ATCGCGCAGGTTCTACTTCG	CGAAGTAGAACCTGCGCGAT
Human LDLRAD3#1	GGTTGTTCCGCTTCCGCTGG	CCAGCGGAAGCGGAACAACC
Human LDLRAD3#2	AATGACGGAGCTGCCGATGA	TCATCGGCAGCTCCGTCATT
Human LDLRAD4#1	TGGCTCTTCACCGTCGGACA	TGTCCGACGGTGAAGAGCCA
Human LDLRAD4#2	ACTGTCAAATATGGTTCGGT	ACCGAACCATATTTGACAGT
Human VLDLR#1	TGGATTCGATAGACGTCCAC	GTGGACGTCTATCGAATCCA
Human VLDLR#2	GGTATCCGAGACTGTGTCGA	TCGACACAGTCTCGGATACC
Human LDLRAP1#1	CGCCGCCATCAAGAGGATCG	CGATCCTCTTGATGGCGGCG
Human LDLRAP1#2	GCGTCATGCCCAGGTACTTG	CAAGTACCTGGGCATGACGC
Human LRPAP1#1	TGATGTCGCTCAGGTCCGAG	CTCGGACCTGAGCGACATCA
Human LRPAP1#2	CCCGCGAGTACTTGCCGCCG	CGGCGGCAAGTACTCGCGGG
