# Supplementary material for: LDLR is an entry receptor for Crimean-Congo hemorrhagic fever virus
Source: Cell Res. 2024 Jan 5;34(2):140–50. doi: 10.1038/s41422-023-00917-w (PMC10837205; doi:10.1038/s41422-023-00917-w)
Supplement: Supplementary file 5 — Supplementary information, Table S2 [file 41422_2023_917_MOESM5_ESM.pdf]

Supplementary information, Table S2. Sequences of primers used in RT-qPCR experiments

Gene	Forward sequence (5'→3')	Reverse sequence (5'→3')
Human *GAPDH*	GAGTCAACGGATTTGGTCGT	GACAAGCTTCCCGTTCTCAG
CCHFV *S*	TAAGCCACAAAGTCCTCGCA	GCCACCTCTGTTGAGAATCAG
RVFV *M*	TCTTGTGATGCCGCCTTCTT	TGTGGGCAGAGAGAGATCCA
EBIV *S*	TTTTGGGTCCATCTCTTTCCTCTGC	GAAAAATGGCATCACCTGGGAAAGT
VSV *L*	ATTCTCTTCCGATTCCCTCG	CCAACTTCCCATCCATTTATGC
SFV *NSP1*	CCGGAGGACGCACAGAAGTTG	TGCGACGGCCACAATCGGAAG
Mouse *Gapdh*	ACGGCCGCATCTTCTTGTGCA	ACGGCCAAATCCGTTCACACC
