# Supplementary material for: LDLR is an entry receptor for Crimean-Congo hemorrhagic fever virus
Source: Cell Res. 2024 Jan 5;34(2):140–50. doi: 10.1038/s41422-023-00917-w (PMC10837205; doi:10.1038/s41422-023-00917-w)
Supplement: Supplementary file 6 — Supplementary information, Table S3 [file 41422_2023_917_MOESM6_ESM.pdf]

Supplementary information, Table S3. siRNA sequences

siRNA	Target sequence (5'→3')
Human LDLR	GGCGTCTCTTCCTATGACA
Human LDLRAP1	CAGACAAGATGCACGACAA
Human LDLRAD1	CTTCCTTGTGGCCCACTGT
Human LDLRAD2	TCCGCTTCTTCCTGGTCTA
Human LDLRAD3	CACGGAATCTCTGAACCAA
Human LDLRAD4	CCCATCAAAGGCAAAGATA
Human LRP1	GGAGTGGTATTCTGGTATA
Human LRP1B	GGATTATTGTAGCAATCAT
Human LRP2	GGAGAAGTATGGAAACAAA
Human LRP3	GTGGTGACATGATTACCAT
Human LRP4	GGATTGAGGTGGCCAATCT
Human LRP5	GGACCAACAGAATCGAAGT
Human LRP6	GCAGAGGCGTAGCATTGAA
Human LRP8	CGAGGACGATGACTGCTTA
Human LRP9	CAAGGAGTCTACATTGCTA
Human LRP10	CTAGAGCCATCACTATTGT
Human LRP11	CAAGCCACCTGCATTATCA
Human LRP12	GCAAGTTCCTCAACTCAGA
Human LRPAP1	GAAGGAAGCGAGACTCATA
Human VLDLR	GTGCAACAATGGCCAGTGT
